# Narrative Review of Clinical Productivity and Teaching in Emergency Medicine

**DOI:** 10.7759/cureus.14309

**Published:** 2021-04-05

**Authors:** Matthew D Zuckerman, Sophia Lin, Fawziah Alsalmi, Simiao Li-Sauerwine

**Affiliations:** 1 Emergency Medicine, University of Colorado School of Medicine, Denver, USA; 2 Department of Emergency Medicine, Weill Cornell Medicine, New York, USA; 3 Department of Emergency Medicine, King Fahad Armed Forces Hospital, Jeddah, SAU; 4 Department of Emergency Medicine, The Ohio State University, Columbus, USA

**Keywords:** education, medical training, workload, teaching, emergency medicine, emergency medicine resident, productivity

## Abstract

Emergency medicine educators are subject to external pressures to increase clinical productivity while maintaining quality teaching. Strategies to mitigate this perceived conflict include alterations in staffing and incentive compensation with educational value units. There is a paucity of information describing the effect of clinical demands on teaching metrics in emergency medicine. We performed a narrative review of the literature describing the relationship between clinical productivity and teaching evaluations of emergency medicine faculty and residents. We searched PubMed and Google Scholar for peer-reviewed articles describing emergency medicine clinical productivity metrics, teaching metrics, and the relationship between them. Seven articles met inclusion criteria. While most articles utilized relative value units (RVUs) per hour, other outcomes metrics were heterogeneous. Almost all studies utilized retrospective data and took place at academic teaching hospitals. Despite variability in statistical analysis, no studies found a relationship between clinical productivity and teaching metrics. Multiple articles identified characteristics of faculty that were associated with improved teaching metrics independent of clinical demands. The available literature does not support the concept that increased clinical productivity conflicts with quality teaching. A subset of faculty was identified who excelled at both. Next research steps should include developing shared standards for assessment of clinical productivity and educational quality that can be used to collect data at multiple sites at academic and community clinical settings; a secondary outcome includes measuring the effects of additional teaching attendings and educational value units.

## Introduction and background

Emergency medicine providers face ever-growing pressure to increasing clinical productivity, leading to a pressing concern that they will have less time to teach and supervise residents and students [1,2]. Simultaneously, providers face an increased workload due to overcrowding from the overall volume, boarding patients, increased administrative burden, time spent documenting, and insufficient staffing [3-5]. In an effort to protect time dedicated to teaching from the encroaching demands of clinical productivity, some academic emergency departments have instituted initiatives to incentivize time spent teaching. These include dedicated teaching shifts and the introduction of educational value units, a metric modeled after the relative value unit (RVU) aimed at quantifying faculty teaching efforts [6-9]. These initiatives were borne out of the perception that clinical productivity and effective bedside teaching are competing demands at odds with one another; however, there is a paucity of data on whether these interventions are necessary or effective [8,10-12].

Several studies have examined whether an increased focus on billing, documentation, and patient satisfaction adversely affects the quality of education in the emergency department. However, these studies are fairly heterogeneous and do not directly address whether the quality of resident education impacts clinical productivity [1,13-16]. Additionally, we sought to collate the outcomes used by researchers to measure teaching efficacy.

In this study, we performed a narrative literature review to determine what is already known about the relationship between clinical productivity and the effectiveness of educational efforts in the emergency department. This review is fundamental to identifying gaps and determining future research, including how to best allocate resources to improve clinical education for emergency medicine trainees.

## Review

Methods

National Library of Medicine Medical Subject Headings (MeSH) was identified through an informal search of the Medline database as well as examining the MeSH headings of previously known papers on the topic. Search terms included: emergency medicine/education, emergency medicine/statistics and numerical data, teaching/methods, and teaching/statistics and numerical data. The Medline database and Google Scholar were queried. Bibliographies of the resulting articles were also examined for previously unidentified studies. Experts in emergency medicine medical education from the Academic Life in Emergency Medicine Faculty Incubator group were consulted via Slack to identify additional articles.

Articles that addressed emergency medicine graduate and undergraduate education and that utilized measurements of clinical productivity and educational quality were included. Articles that focused on fields outside emergency medicine were excluded. While the English language was not a requirement, no identified articles required translation. Each article was reviewed by at least two researchers and the results were entered into a shared spreadsheet. This research was exempt from review by the Colorado Multiple Institutional Review Board.

Results

Seven articles were identified (Table 1). One study was excluded as it examined the relationship between resident productivity and teaching of medical students. Two studies prospectively collected data, while the remaining studies used retrospectively collected data [13,15]. While the response rate was not regularly reported, the response rate in the prospective study was 89%. Our study protocol was successful at identifying appropriate literature.

**Table 1:** Primary studies of clinical productivity and educational metrics ED: emergency department, RVU: relative value unit.

Primary Author and Year	Population and Design	Intervention	Measured Outcome(s)	Main Findings
Kelly et al. [13]	Urban academic ED, prospective observational	Some shifts had additional teaching attending	Patients/hour; resident perception of workload; attending perception of workload; attending workload; attending teaching evaluation	The increased workload does not affect clinical teaching scores. Clinical teaching skills, willingness to teach, and learning environment are more important. The presence of teaching attending did not affect overall teaching scores.
Clyne et al. [16]	Urban academic ED, retrospective observational		RVUs/hour; patients/hour; hours at primary teaching site; attending teaching evaluation; attending years post-training	No correlation between clinical teaching scores and productivity. An inverse relationship between clinical teaching scores and years post-training.
Hemphill et al. [1]	Academic ED; mixed methods - retrospective data + faculty interviews		RVUs/hour; patient satisfaction scores; attending teaching evaluation	Higher resident evaluation scores correlate with higher productivity. No correlation between medical student evaluation scores and productivity.
Colletti et al. [17]	Urban academic ED, retrospective observational		Median patient throughput time; five educational domains were modeled on responses to 18 question survey	Openness and enthusiasm associated with a shorter patient visit; commitment to knowledge and instruction was associated with a longer patient visit
Berger et al. [15]	Prospective, multisite including inner-city public hospital, academic community hospital, tertiary care university hospital		RVU/hour; medical student post-shift survey of attending teaching	No significant relationship between clinical productivity and medical students’ perception of quality of teaching in the ED
Begaz et al. [14]	Single academic center, retrospective		RVU/hour; average annual length of stay; attending teaching evaluations by residents and medical students	No relationship between clinical effectiveness and teaching scores

Sites

All studies included were conducted at urban academic emergency departments. Berger et al. conducted their study at three academic emergency departments including an inner-city public hospital and an academic center-affiliated community hospital [15]. All other studies were single-site studies. Patient volume at the emergency departments where these studies were conducted ranged from 25,000 visits/year to 100,000 visits/year.

Interventions

One study noted that the presence of an additional faculty member for teaching did not have an impact on teaching scores. None of the articles evaluated the impact of educational value units.

Strategies

Hemphill et al. included a semi-structured interview with faculty who performed well on both clinical and teaching metrics [1]. They believed that faculty-identified specific strategies contributed to their success. Strategies for maintaining teaching scores included teaching on every patient and discussing decision rules and medical decision making. While some faculty attempted to maintain productivity metrics by seeing a minimum number of patients per hour, others maintained RVUs by ensuring complete charting on every patient. Despite such high performance, all faculty interviewed “believe that as the emphasis on billing productivity increases, resident and student education will suffer.”

Clinical Metrics

The most commonly used metric for evaluating physician productivity was RVUs generated per hour. Berger et al. used this metric as their sole measure of clinical productivity while three studies used this metric in combination with other measures [14-17]. Two studies examined throughput time: Colletti et al. studied throughput time for all patients while Begaz et al. studied throughput time for only discharged patients [14,17]. Two studies included the number of patients seen per hour. One study examined the percentage of post-ED visit patient surveys in which the attending physician was rated as “excellent” as a measure of patient satisfaction [17]. In addition to using patients seen per hour, Kelly et al. also used both resident and attending perception of shift workload as markers for clinical productivity, which had some agreement [13].

Teaching Metrics

Five studies utilized resident evaluations to measure educational productivity [1,13,14,16,17]. In four of these studies, residents completed evaluations either annually or semi-annually. In the fifth study in which data were collected prospectively, residents were surveyed at the end of every shift when a research associate was available [13]. In addition to using resident evaluation scores as a teaching metric, Hemphill et al. collected faculty ratings by medical students at the end of their required emergency medicine rotation [1]. Berger et al. utilized only medical student evaluations completed at the end of a shift as their primary outcome measure for teaching effectiveness [15].

Hemphill et al. further probed the means by which faculty achieved both high clinical and educational metrics by interviewing attending physicians who generated high RVUs and obtained high scores on resident evaluations [1]. As discussed above, the faculty identified several strategies for teaching on shift: teaching on every patient, exploring clinical decision rules, and guiding residents through medical decision making.

Colletti et al. had the most complex modeling of teaching metrics, using a multivariate model to translate survey responses into five domains of instructional quality [17].

Study Limitations

Most studies were single-site studies and all studies occurred at large academic teaching hospitals. Only one study included an academically affiliated community hospital site [15]. Thus, the results of these studies may not be broadly generalizable. Additionally, except for one study, data on resident teaching metrics were collected either annually or semi-annually, making a correlation between teaching performance averaged over the year and clinical productivity by shift unfeasible [13].

Two studies noted that resident evaluations were anonymous and one study noted that medical student evaluations were anonymous [14-16]. In the remaining studies, it is unclear whether or not trainee evaluations were anonymous or identifiable. Evaluations by learners which identify the writer have been shown to lead to inflated teaching scores [18]. However, any skew in faculty educational scores should be consistent within one institution.

All studies utilized Likert scale-based evaluations to assess faculty teaching performance. While teaching evaluations frequently allow for descriptive comments, none of the studies indicated the existence of such commentary. Analysis of teaching metrics in all of the studies focused on numerical scores. It is possible that the inclusion of qualitative statements about educators could provide additional information.

Discussion

The body of literature on the relationship between clinical productivity and teaching scores is scant, despite the degree of concern that emphasis on clinical productivity metrics negatively impacts educational efforts in the ED. While outcomes were heterogeneous, a recurrent finding was that teaching scores did not inversely correlate with clinical productivity measures. Some investigators found that a subset of faculty excelled in both clinical performance and teaching. Individuals seeking to excel at teaching on shift should teach on every patient and focus on decision rules and medical decision making. Kelly et al. found that resident perception of teaching quality was associated with specific faculty characteristics rather than perceived clinical workload [13]. This may be because attributes such as energy, enthusiasm, and practicality positively impact clinical productivity as well as teaching effectiveness. These traits may allow a clinician to work efficiently in the ED while effectively educating trainees concomitantly.

Within existing literature, there is a variation on how clinical productivity is measured: RVU, throughput time, number of patients seen per hour, perception of clinical workload, and patient satisfaction. The efficacy of teaching is measured primarily by scores and feedback provided by residents or infrequent faculty evaluations. These are often based on similar concepts, but the exact questions and scales can vary between departments.

Whatever survey instrument is used, prospectively collected data are one approach to better match outcome measures on a given shift. Alternatively, having residents link their faculty feedback to a particular shift could also allow for matching of educational metrics with shift clinical metrics.

None of the articles addressed the impact of educational value units on clinical or educational metrics. The addition of teaching attending is another way to increase resources for on-shift teaching, but Kelly et al. did not find improved outcomes [13]. 

Future Directions

Future studies would benefit from collecting data from a breadth of practice settings including more data from community or community-affiliated academic sites. Additionally, prospective data collection would allow for the examination of shift-to-shift variability in RVUs and learner perceptions. Future studies should include sites with educational value units and assigned teaching attendings. Such interventions can be costly, and evaluating their efficacy is important for deciding on resource allocation.

A study that does not find a relationship between clinical demands and education might not help to alleviate faculty anxiety about time for teaching. Studies that include structured interviews of high performers and direct observation of faculty behaviors may identify strategies that help physician educators to excel at both productivity and education. Additionally, as most departments collect similar data on clinical and educational productivity, a multisite analysis may provide further data. Such analysis could also help examine proposed interventions such as educational value units and protected time.

Identification of faculty who are high performing in both clinical and educational productivity may identify strategies and characteristics that would improve resident teaching evaluations overall.

Limitations

This review has several important limitations. We relied on studies indexed in Medline and Google Scholar. We surveyed a small number of experts to identify additional articles. We did not formally assess articles for quality. We excluded an article that specifically focused on resident productivity.

## Conclusions

In conclusion, while emergency medicine physician educators will likely continue to express concerns over how clinical demands will affect teaching, the current data does not bear this out. Future study should focus on increasing the quantity and quality of data available. Additionally, qualitative methods and subgroup analysis of high performers may identify teaching and clinical strategies that can be implemented. The importance of this issue suggests that a systematic research agenda may accelerate the process.
